# Adherence to Eye Examination Guidelines Among Individuals With Diabetes in Saudi Arabia

**DOI:** 10.7759/cureus.51472

**Published:** 2024-01-01

**Authors:** Haneen O Alhujaili, Afaf M Alanazi, Waleed M Alshehri, Rayan A Alghamdi, Abdulmlk A Alqasem, Fatima I Alhumaid, Rozan A AlGhamdi, Khalid K Almaymuni

**Affiliations:** 1 Ophthalmology, Ohod General Hospital, Al Madina, SAU; 2 Medicine, Qassim University, Buraydah, SAU; 3 General Practice, King Salman Bin Abdulaziz Medical City, Madina, SAU; 4 Medicine and Surgery, Imam Mohammad Ibn Saud Islamic University, Riyadh, SAU; 5 Ophthalmology, Al Jabr Eye and ENT Hospital, Al Ahsa, SAU; 6 Faculty of Medicine, King Abdulaziz University, Jeddah, SAU; 7 General Practice, King Fahad Specialist Hospital, Buraydah, SAU

**Keywords:** eye, preventive strategies, adherence, diabetes, diabetic retinopathy

## Abstract

Background: Individuals with diabetes are at risk of developing diabetic retinopathy, a vision-threatening complication. Regular eye examinations are crucial for early detection and management. Ensuring adherence to eye examination guidelines is essential to prevent visual impairment and blindness in this at-risk population.

Methods: This was cross-sectional study, and a validated questionnaire was physically administered among adult individuals with diabetes (both males and females) in Saudi Arabia. The data were gathered and analyzed using Statistical Product and Service Solutions (SPSS, version 26.0) software (IBM SPSS Statistics for Windows, Armonk, NY). This study took approximately three months from the period August 2023 to November 2023.

Results: This study found that most participants had type 1 diabetes (46.1%, n=83) and had been diagnosed for more than 10 years (49.4%, n=89). The most used management strategy (48.9%, n=88) was lifestyle changes and anti-diabetic medications. Most of the participants (93.3%, n=168) were fully aware of the severe eye complications of diabetes, as well as diabetic retinopathy and its complications (48.9%, n=88). The most common source of information about the importance of eye exams was healthcare professionals (56.7%, n=102). Most of the participants had annual eye exams (58.3%, n=105) and within the previous year, specifically related to their diabetes (62.8%, n=113). Adherence to eye examination guidelines was higher in those who had diabetes for more than 10 years (p=0.009), those who were on lifestyle changes and insulin therapy or anti-diabetic medications (p=0.030), those who were fully aware of severe eye complications and diabetic retinopathy (p=0.017 and p=0.020, respectively), and those with type 2 diabetes (p=0.001). In addition, participants who understood the importance of eye examinations had better glucose control (p=0.017), had eye examinations within the previous year (p=0.001), and had heard about the importance of eye examinations from healthcare professionals (p=0.020). The findings revealed the most common reasons for not getting an eye exam were a lack of awareness (37.8%) and distance from the hospital.

Conclusion: To summarize, many people with diabetes do not get regular eye exams often because they are unaware of how important these exams are. Long-term diabetics who are aware of the dangers of diabetic retinopathy are more likely to heed this advice. However, adherence was linked to more frequent eye exams and better glucose control. Adherence and wide awareness must be created to improve retinopathy outcomes.

## Introduction

Diabetes, a chronic metabolic disorder, is on the rise globally, significantly impacting developing nations such as Saudi Arabia. According to the World Health Organization, Saudi Arabia ranks among the highest in the prevalence of diabetes mellitus (DM), standing as the second-highest in the Middle East, with over seven million diagnosed cases of DM and three million individuals classified as pre-diabetics [1,2]. This condition is characterized by elevated blood glucose levels due to insufficient insulin production, leading to a wide array of complications affecting multiple body systems [3]. Among these complications, diabetic retinopathy stands out as a primary cause of blindness among the adult population worldwide [4].

According to the World Health Organization, it is estimated around one-third of patients with DM have diabetic retinopathy changes and need to have screening every one to two years, and the interval of screening might be adjusted according to the glycemic control with a maximum interval of two years [5]. This sheds light on the clinical importance of regular eye examination for those people, with diabetes to ensure early detection and proper intervention [6]. In Saudi Arabia, the occurrence of diabetic retinopathy is estimated at 34.6% among individuals diagnosed with diabetes, a notably high rate in contrast to the global prevalence of 27% [7]. The sedentary lifestyle and dietary habits in the country have contributed largely to this health crisis [8]. With that being said, the efficacy of preventive measures such as routine eye examinations is of crucial importance [9].

Previous research has shown variety in adherence to recommended eye examination guidelines among diabetics. These studies outline a variety of barriers to eye care adherence, including the lack of awareness and eye care service access [10]. However, not enough data were published regarding this in Saudi Arabia, underscoring the need for research exploring this issue within the Saudi population [11]. This cross-sectional study aims to evaluate how well diabetic individuals in Saudi Arabia adhere to eye examination guidelines. These guidelines are outlined in the 2018 Standards of Medical Care in Diabetes by the American Diabetes Association. At 34.6% of diabetic patients nationwide, diabetic retinopathy has a frighteningly high prevalence [12]. Lifestyle elements that contribute to this health crisis include diet and sedentary behavior. Prior research has indicated obstacles to upholding adherence to eye care, such as low awareness and restricted access to eye care services [13].

This study will shed light into the effectiveness of current preventive strategies, obstacles faced, and potential strategies for improvement. As a result, this could contribute to the formation of policies and the development of interventions that encourages adherence to these guidelines, for an ultimate aim of lessening the impact of diabetic retinopathy within the Saudi population. The hope that this research will have a positive impact on enhancing the quality of life of individuals with diabetes in Saudi Arabia and globally.

## Materials and methods

Study design: A descriptive cross-sectional study was conducted among the adult diabetic patients to determine their adherence to eye examination guidelines from 1 August and 3 November 2023. The population included patients from outpatient departments from primary health centers in Riyadh and Qassim and those at King Khalid Hospital in Hail.

Sample size: The minimum acceptable sample size was determined using the formula ss=(Z2pq)/c2, where ss=sample size, Z=1.96, p=0.5, q=(1-p)=0.5, and c=sampling error of 8%. This study, hence, adopted a sample of 180 diabetic patients.

Sampling technique: The subjects were selected through convenience sampling techniques as it was the most appropriate technique to sample our population of interest, the diabetic patients.

Study population, inclusion, and exclusion: This study included adult diabetic patients’ centers mentioned above. The inclusion criteria for this study encompass all adult diabetic patients, including both T1DM and T2DM, regardless of gender, but residing within Saudi Arabia. On the other hand, exclusion criteria encompass individuals with gestational diabetes, non-diabetic patients, and diabetic patients residing outside the boundaries of Saudi Arabia. Additionally, since informed consent was an imperative ethical consideration, we excluded the patients that were unwilling to participate.

Data collection methods: Data were collected through a validated questionnaire and issued to the target population. The questionnaire was administered to the patients with the help of caregivers after acquiring permission from the authorities in charge. The questionnaire was made up of three key sections. The first section involved social demographic data, the second section involved items clinical characteristics, and the third section has items on awareness.

Data analysis: The data collected were entered and cleaned using Microsoft Excel and analyzed using the Statistical Product and Service Solutions (SPSS, version 26; IBM Corp., Armonk, NY). Frequencies and percentage were obtained using descriptive statistics. Inferential statistics were also obtained using the chi-square test. P-value was statistically significant at p<0.05.

Ethical considerations: Ethical approval for this study was obtained from the Committee of Research Ethics at Qassim University. As a result, our investigation was approved with IRB approval number 23-60-6. Informed consent and confidentiality were some of the ethical considerations. The respondents were assured that they had the right to withdraw consent and abandon the process at any point during the survey.

## Results

Table 1 shows that most of the respondents, 37.8% (n=68), were between the ages 18 and 45. The second largest age group consisted of individuals between the ages of 46 and 60, accounting for 35.0% (n=63) of the total sample. There were 27.2% of the participants over 60 years (n=49). The gender distribution of the sample was 38.9% (n=70) consisting of male and 61.1% (n=110) female. The largest group in terms of education was represented by those with a bachelor's degree, accounting for 49.3% (n=89) of the sample. The next group, comprising 25.6% (n=46), were those who had finished high school. The smallest group was made up of people who had only primary school education (5.6%, n=10). Regarding employment, the greatest category consisted of individuals who had no job, making up 46.1% of the sample (n=83). Those employed in the education sector came next, making up 28.3% (n=51) of the sample. The health sector (6.7%, n=12), the military sector (2.8%, n=5), and other sectors (16.1%, n=29) comprised the remaining occupations. Regarding smoking status, 87.2% of respondents (n=157) indicated that they do not smoke, whereas 12.8% of respondents (n=23) were smokers.

**Table 1:** Demographic characteristics of the participant (n=180) Data have been represented as n and %.

Social characteristics	Frequency and percentage (%)
AGE	
18-45 years	68 (37.8%)
60-46	63 (35.0%)
Above 60	49 (27.2%)
Gender	
Male	70 (38.9%)
Female	110 (61.1%)
Education level	
Bachelor	89 (49.3%)
Elementary school	12 (6.7%)
High school	46 (25.6%)
Other	23 (12.8%)
Primary school	10 (5.6%)
Occupation	
Education	51 (28.3%)
Health field	12 (6.7%)
Military field	5 (2.8%)
Not working in any job	83 (46.1%)
Other	29 (16.1%)
Smoking status	
Yes	23 (12.8%)
No	157 (87.2%)

Table 2 shows that 46.1% (n=83) of the participants had T1DM, and 53.9% (n=97) had T2DM. In terms of the length of the diabetes diagnosis, most of the participants 49.4% (n=89) had been diagnosed for more than 10 years. Regarding diabetes management, 48.9% (n=88) used lifestyle modifications and anti-diabetic medications. In terms of controlling glucose readings, 61.7% (n=111) were able to control within the reading range all or most of the time. More so, most of the participants (93.3%, n=168) were fully aware that diabetes can cause severe eye complications. Further, most of the respondents 48.9% (n=88) were fully aware of diabetic retinopathy and its complications. The most common source of information about importance of eye examinations for individuals with diabetes was healthcare professionals (doctors and nurses) (56.7%, n=102), followed by internet research or social media platforms (17.2%, n=31), friends or family with diabetes (16.1%, n=29), and diabetes public campaigns or organizations (10%, n=18). In terms of the frequency of eye examinations, most of the participants (58.3%, n=105) underwent eye examinations every year. Additionally, most of the participants (62.8%, n=113) had an eye examination specifically related to their diabetes within the past year. Finally, most of the participants (37.8%, n=68) indicated the reason for not going for the eye examination is that they were not aware of the importance of eye checkups.

**Table 2:** Prevalence of diabetes awareness among the participants (n=180) Data have been represented as n and %.

Variables	Frequency (n) and proportion (%)
Type of DM	
Type 1	83 (46.1%)
Type 2	97 (53.9%)
Length of DM diagnosis	
Five to ten years	42 (23.3%)
Less than 5 years	49 (27.2%)
More than 10 years	89 (49.4%)
Diabetes mellitus management plan	
Lifestyle modifications only	16 (8.9%)
Lifestyle modifications and anti-diabetic medications	88 (48.9%)
Lifestyle modification and insulin therapy	76 (42.2%)
Control of glucose reading within the reading range	
Yes, all or most of the time	111 (61.7%)
Sometimes	58 (32.2%)
Rarely	11 (6.1%)
Awareness of diabetes can cause severe eye complications	
Yes, I'm fully aware	168 (93.3%)
Somewhat aware	11 (6.1%)
Not aware at all	1 (0.6%)
Knowledge of diabetes retinopathy and its complications	
Yes, I'm fully aware	88 (48.9%)
Somewhat aware	58 (32.2%)
Not aware at all	34 (18.9%)
Sources information about the importance of eye examinations for individuals with diabetes	
Healthcare professionals (doctors, nurses)	102 (56.7%)
Diabetes public campaigns or organizations	18 (10%)
internet research or social media platforms	31 (17.2%)
Friends or family with diabetes	29 (16.1%)
Frequency of individuals with diabetes type 2 undergoing eye examinations	
Every year	105 (58.3%)
Every 3 year	5 (2.8%)
Only if experiencing vision problems	19 (10.6%)
Not sure	48 (26.7%)
Ever had an eye examination specifically related to your diabetes	
Yes, within the past year	113 (62.8%)
Yes, more than a year ago	25 (13.9%)
No, never had one	42 (23.3%)
Reasons for not having the eye exam	
Eye checkups are very costly	18 (10.0%)
I’m not aware of the importance of eye check up	68 (37.8%)
Hospitals are far away from my home	24 (13.3%)
I’m afraid of knowing that I’m going blind	10 (5.6%)
No valid reason	60 (33.3%)

Table 3 shows that individuals who have had diabetes for more than 10 years are more likely to follow eye examination guidelines than those who have had it for less than 10 years (p=0.009). Adherence was significantly improved by the management plan. Individuals on insulin therapy and lifestyle changes have the highest adherence, followed by those on lifestyle changes and anti-diabetic medications, while those on lifestyle modifications only have the lowest adherence (p=0.030). Individuals who are fully aware of the severe eye complications of diabetes are more likely than those who are only partially or completely unaware to follow eye examination guidelines (p=0.017). Further, individuals who are fully aware of diabetes retinopathy and its complications are more likely to follow eye examination guidelines than those who are only partially or completely unaware (p=0.020).

**Table 3:** Impact of healthcare provider recommendations and patient education on adherence to eye examination guidelines among individuals with diabetes (n=180) Data have been represented as n and %. A p-value of <0.05 was considered statistically significant.

Variables	Total, n (%)	Diabetes public campaigns or organizations, n (%)	Friends or family with diabetes, n (%)	Healthcare professionals (doctors, nurses), n (%)	Internet research or social media platforms, n (%)	* P-value
Type of DM						0.247
Type 1	83 (46.1%)	10 (12%)	9 (10.8%)	51 (61.4%)	13 (15.7%)	
Type 2	97 (53.9%)	8 (8.2%)	20 (20.6%)	51 (52.6%)	18 (18.6%)	
Length of DM Diagnosis						0.009
Five to ten years	42 (23.3%)	7 (16.7%)	3 (7.1%)	24 (57.1%)	8 (19.0%)	
Less than 5 years	49 (27.2%)	7 (14.3%)	14 (28.6%)	19 (38.8%)	9 (18.4%)	
More than 10 years	89 (49.4%)	4 (4.5%)	12 (13.5%)	59 (66.3%)	14 (15.7%)	
Diabetes mellitus management plan						0.030
Lifestyle modifications only	16 (8.9%)	2 (12.5%)	4 (25%)	5 (31.3%)	5 (31.3%)	
Lifestyle modifications and anti-diabetic medications	88 (48.9%)	8 (9.1%)	19 (21.6%)	44 (50%)	17 (19.3%)	
Lifestyle modification and Insulin therapy	76 (42.2%)	8 (10.5%)	6 (7.9%)	53 (69.7%)	9 (11.8%)	
Control of glucose reading within the reading range						0.366
Yes, all or most of the time	111 (61.7%)	12 (10.8%)	18 (16.2%)	62 (55.9%)	19 (17.1%)	
Sometimes	58 (32.2%)	5 (8.6%)	7 (12.1%)	37 (63.8%)	9 (15.5%)	
Rarely	11 (6.1%)	1 (9.1%)	4 (38.4%)	3 (27.3%)	3 (27.3%)	
Awareness of diabetes can cause severe eye complications						0.017
Yes, I'm fully aware	168 (93.3%)	18 (10.7%)	23 (13.7%)	99 (58.9%)	28 (16.7%)	
Somewhat aware	11 (6.1%)	0 (0.0%)	5 (45.5%)	3 (27.3%)	3 (27.3%)	
Not aware at all	1 (0.6%)	0 (0.0%)	1 (100%)	0 (0.0%)	0 (0.0%)	
knowledge of diabetes retinopathy and its complications						0.020
Yes, I'm fully aware	88 (48.9%)	11 (12.5%)	8 (9.1%)	58 (65.9%)	11 (12.5%)	
Somewhat aware	58 (32.2%)	7 (12.1%)	12 (20.7%)	26 (44.8%)	13 (22.4%)	
Not aware at all	34 (18.9%)	0 (0.0%)	9 (26.5%)	18 (52.9%)	7 (20.6%)	
Frequency of individuals with diabetes type 2 undergoing eye examinations						0.744
Every year	105 (58.3%)	9 (8.6%)	15 (14.3%)	62 (59.0%)	19 (18.1%)	
Every 3 year	5 (2.8%)	0 (0.0%)	1 (20%)	4 (80%)	0 (0.0%)	
Only if experiencing vision problems	19 (10.6%)	4 (21.1%)	3 (15.8%)	9 (47.4%)	3 (15.8%)	
Not sure	48 (26.7%)	5 (10.4%)	10 (20.8%)	24 (50%)	9 (18.8%)	
Ever had an eye examination specifically related to your diabetes						0.125
Yes, within the past year	113 (62.8%)	8 (7.1%)	17 (15.0%)	73 (64.6%)	15 (13.3%)	
Yes, more than a year ago	25 (13.9%)	4 (16%)	3 (12%)	12 (48%)	6 (24%)	
No, never had one	42 (23.3%)	6 (14.3%)	9 (21.4%)	17 (40.5%)	10 (23.8%)	
Reason for not having the eye exam						0.650
Eye checkups are very costly	18 (10.0%)	1 (5.6%)	1 (5.6%)	12 (66.7%)	4 (22.2%)	
I’m not aware of the importance of eye check up	68 (37.8%)	7 (10.3%)	17 (25%)	32 (47.1%)	12 (17.6%)	
Hospitals are far away from my home	24 (13.3%)	3 (12.5%)	2 (8.3%)	16 (66.7%)	3 (12.5%)	
I’m afraid of knowing that I’m going blind	10 (5.6%)	2 (22.2%)	2 (22.2%)	4 (44.4%)	2 (22.2%)	

Table 4 indicates that individuals with T2DM were more likely to adhere to a plan that included anti-diabetic drugs and lifestyle modifications than those with T1DM (78.4% vs. 21.6%, p=0.001). However, compared to people with T2DM, people with T1DM were more likely to adhere to a DM management plan that included insulin therapy and lifestyle modifications (73.7% vs. 26.3%, p=0.001). In addition, individuals with T2DM reported yearly eye exams at a significantly higher rate (57.1% vs. 42.9%, p=0.027) than those with T1DM.

**Table 4:** Comparison of the adherence to eye examination guidelines among individuals with type 1 diabetes and type 2 diabetes (n=180) Data have been represented as n and %. A p-value of <0.05 was considered statistically significant.

Variables	Total, n (%)	Diabetes, type 1, n (%)	Diabetes, type 2, n (%)	*P-value
Length of DM diagnosis				0.676
Five to ten years	42 (23.3%)	18 (42.9%)	24 (57.1%)	
Less than 5 years	49 (27.2%)	21 (42.9%)	28 (57.1%)	
More than 10 years	89 (49.4%)	44 (49.4%)	45 (50.6%)	
Diabetes mellitus management plan				0.001
Lifestyle modifications only	16 (8.9%)	8 (50%)	8 (50%)	
Lifestyle modifications and anti-diabetic medications	88 (48.9%)	19 (21.6%)	69 (78.4%)	
Lifestyle modification and Insulin therapy	76 (42.2%)	56 (73.7%)	20 (26.3%)	
Control of glucose reading within the reading range				0.172
Yes, all or most of the time	111 (61.7%)	57 (51.4%)	54 (59.8%)	
Sometimes	58 (32.2%)	21 (36.2%)	37 (63.8%)	
Rarely	11 (6.1%)	5 (45.5%)	6 (54.5%)	
Awareness of diabetes can cause severe eye complications				0.092
Yes, I'm fully aware	168 (93.3%)	80 (47.6%)	88 (52.4%)	
Somewhat aware	11 (6.1%)	2 (18.2%)	9 (81.8%)	
Not aware at all	1 (0.6%)	1 (100%)	0 (0.0%)	
Knowledge of diabetes retinopathy and its complications				0.491
Yes, I'm fully aware	88 (48.9%)	83 (46.1%)	97 (53.9%)	
Somewhat aware	58 (32.2%)	26 (44.8%)	32 (55.2%)	
Not aware at all	34 (18.9%)	13 (38.2%)	21 (61.8%)	
Sources information about the importance of eye examinations for individuals with diabetes				0.247
Healthcare professionals (doctors, nurses)	102 (56.7%)	51 (50%)	51 (50%)	
Diabetes public campaigns or organizations	18 (10%)	10 (55.6%)	8 (44.4%)	
Internet research or social media platforms	31 (17.2%)	13 (41.9%)	18 (58.1%)	
Friends or family with diabetes	29 (16.1%)	9 (31.0%)	20 (69.0%)	
Frequency of individuals with diabetes type 2 undergoing eye examinations				0.027
Every year	105 (58.3%)	45 (42.9%)	60 (57.1%)	
Every 3 year	5 (2.8%)	4 (80%)	1 (20%)	
Only if experiencing vision problems	19 (10.6%)	4 (21.1%)	15 (78.9%)	
Not sure	48 (26.7%)	28 (53.3%)	20 (41.7%)	
Ever had an eye examination specifically related to your diabetes				0.125
Yes, within the past year	113 (62.8%)	57 (50.4%)	56 (49.6%)	
Yes, more than a year ago	25 (13.9%)	7 (28.0%)	25 (72%)	
No, never had one	42 (23.3%)	19 (45.2%)	23 (54.8%)	
Reason for not having the eye exam				0.443
Eye checkups are very costly	18 (10.0%)	10 (55.6%)	8 (44.4%)	
I’m not aware of the importance of eye checkup	68 (37.8%)	28 (41.2%)	40 (58.8%)	
Hospitals are far away from my home	24 (13.3%)	9 (37.5%)	15 (62.5%)	
I’m afraid of knowing that I’m going blind	10 (5.6%)	6 (60%)	4 (40%)	

Table 5 shows that following eye examination guidelines was associated with better diabetic retinopathy outcomes. Participants who reported being fully aware of the importance of eye examinations were more likely to have better control of glucose readings within the recommended range (56.8% vs 36.2%, p=0.017). They were also more likely to have had an eye examination specifically related to their diabetes within the previous year (62.8% vs 40.0% for those who were not aware, p=0.001). Participants who reported being fully aware of the importance of eye examinations were more likely to have heard about it from healthcare professionals (56.7% vs 17.6% for those who were somewhat or not at all aware, p=0.020). Individuals with T2DM who had an eye examination every year were more likely to understand the importance of eye examinations. Individuals with T2DM had a higher frequency of undergoing eye examination with 64 (61%) of them doing it every year. The was a statistically significant relationship between awareness of the diabetic condition and the frequency of the eye checkup. Reasons for not having an eye examination included a lack of awareness about the importance of eye exams (37.8%) and hospitals being too far away from their home (13.3%). These results were statistically significant with p-value <0.05.

**Table 5:** Association between adherence to eye examination guidelines and diabetic retinopathy outcomes among individuals with diabetes (n=180) Data have been represented as n and %. A p-value of <0.05 was considered statistically significant.

Variables	Total, n (%)	Yes am fully aware, n (%)	Somewhat aware, n (%)	Not aware at all, n (%)	*P-value
Type of DM					0.491
Type 1	83 (46.1%)	13 (15.7%)	26 (31.3%)	44 (40.6%)	
Type 2	97 (53.9%)	21 (21.6%)	32 (33.0%)	44 (45.4%)	
Length of DM diagnosis					0.424
Five to ten years	42 (23.3%)	9 (21.4%)	16 (38.1%)	17 (40.5%)	
Less than 5 years	49 (27.2%)	12 (24.5%)	15 (30.6%)	22 (44.9%)	
More than 10 years	89 (49.4%)	13 (14.6%)	27 (30.3%)	49 (55.1%)	
Diabetes mellitus management plan					0.36
Lifestyle modifications only	16 (8.9%)	1 (6.3%)	4 (25%)	11 (68.8%)	
Lifestyle modifications and anti-diabetic medications	88 (48.9%)	22 (25%)	33 (37.5%)	33 (37.5%)	
Lifestyle modification and Insulin therapy	76 (42.2%)	11 (14.5%)	21 (27.6%)	44 (57.9%)	
Control of glucose reading within the reading range					0.017
Yes, all or most of the time	111 (61.7%)	13 (11.7%)	35 (31.5%)	63 (56.8%)	
Sometimes	58 (32.2%)	17 (29.3%)	20 (34.5%)	21 (36.2%)	
Rarely	11 (6.1%)	4 (36.4%)	3 (27.3%)	4 (36.4%)	
Awareness of diabetes can cause severe eye complications					0.001
Yes, I'm fully aware	168 (93.3%)	27 (16.1%)	53 (31.5%)	88 (52.4%)	
Somewhat aware	11 (6.1%)	6 (54.5%)	5 (45.5%)	0 (0.0%)	
Not aware at all	1 (0.6%)	1 (100%)	0 (0.0%)	0 (0.0%)	
Sources information about the importance of eye examinations for individuals with diabetes					0.020
Healthcare professionals (doctors, nurses)	102 (56.7%)	18 (17.6%)	26 (25.5%)	58 (56.9%)	
Diabetes public campaigns or organizations	18 (10%)	0 (0.0%)	7 (38.9%)	11 (61.1%)	
Internet research or social media platforms	31 (17.2%)	7 (22.6%)	13 (41.9%)	11 (35.5%)	
Friends or family with diabetes	29 (16.1%)	9 (31%)	12 (41.4%)	8 (27.6%)	
Frequency of individuals with diabetes type 2 undergoing eye examinations					0.001
Every year	105 (58.3%)	11 (10.5%)	30 (28.6%)	64 (61%)	
Every 3 year	5 (2.8%)	1 (20%)	1 (20%)	3 (60%)	
Only if experiencing vision problems	19 (10.6%)	3 (15.8%)	9 (47.4%)	7 (36.8%)	
Not sure	48 (26.7%)	19 (39.6%)	17 (35.4%)	12 (25%)	
Ever had an eye examination specifically related to your diabetes					0.001
Yes, within the past year	113 (62.8%)	12 (10.6%)	32 (28.3%)	68 (61.8%)	
Yes, more than a year ago	25 (13.9%)	5 (20%)	10 (40%)	10 (40%)	
No, never had one	42 (23.3%)	17 (40.5%)	16 (38.1%)	9 (21.4%)	
Reason for not having the eye exam					0.022
Eye checkups are very costly	18 (10.0%)	3 (16.7%)	9 (50.0%)	6 (33.3%)	
I’m not aware of the importance of eye check up	68 (37.8%)	21 (30.9%)	20 (29.4%)	27 (39.7%)	
Hospitals are far away from my home	24 (13.3%)	3 (12.5%)	7 (29.2%)	14 (58.3%)	
I’m afraid of knowing that I’m going blind	10 (5.6%)	2 (20%)	4 (40%)	4 (40%)	

## Discussion

Diabetic retinopathy is a prevalent complication in both T1DM and T2DM due to retinal microvasculature damage, affecting approximately 27% of the global diabetic population. It stands as a primary cause of vision loss in individuals aged in diabetic patients and can be prevented up to 90% through early intervention. Ensuring adherence to eye examination in their initial stages can facilitate prompt treatment, preventing further deterioration and potential vision loss [14]. This study aimed to evaluate diabetic patients' adherence to eye examination in Saudi Arabia while pinpointing their level of awareness and practices on eye examinations.

In addition, the study findings revealed that 46.1% (n=83) of the participants had T1DM, while 53.9% (n=97) had T2DM. In terms of the length of the diabetes diagnosis, most of the participants (49.4%, n=89) had been diagnosed for more than 10 years. Regarding diabetes management, 48.9% (n=88) used lifestyle modifications and anti-diabetic medications. In terms of controlling glucose readings, 61.7% (n=111) were able to control within the reading range all or most of the time. More so, most of the participants (93.3%, n=168) were fully aware that diabetes can cause severe eye complications. Further, most of the respondents (48.9%, n=88) were fully aware of diabetic retinopathy and its complications. The most common source of information about importance of eye examinations for individuals with diabetes was healthcare professionals (doctors and nurses) (56.7%, n=102), followed by internet research or social media platforms (17.2%, n=31), friends or family with diabetes (16.1%, n=29), and diabetes public campaigns or organizations (10%, n=18). In terms of the frequency of eye examinations, most of the participants (58.3%, n=105) underwent eye examinations every year. Additionally, most of the participants (62.8%, n=113) had an eye examination specifically related to their diabetes within the past year. Finally, most of the participants (37.8%, n=68) indicated the reason for not going for the eye examination is that they were not aware of the importance of eye checkups. These findings are consistent with the findings by Schoenfeld et al. in their study, which indicated that less than half of participants had annual eye examinations based on the duration of diabetic condition of the participants [15].

On the other hand, the study revealed that individuals who have had diabetes for more than 10 years are more likely to follow eye examination guidelines than those who have had it for less than 10 years (p=0.009). Adherence was significantly improved by the management plan. Individuals on insulin therapy and lifestyle changes have the highest adherence, followed by those on lifestyle changes and anti-diabetic medications, while those on lifestyle modifications only have the lowest adherence (p=0.030). Individuals who are fully aware of the severe eye complications of diabetes are more likely than those who are only partially or completely unaware to follow eye examination guidelines (p=0.017). Further, individuals who are fully aware of diabetes retinopathy and its complications are more likely to follow eye examination guidelines than those who are only partially or completely unaware (p=0.020). More so, the study found that individuals with T2DM were more likely to adhere to a plan that included anti-diabetic drugs and lifestyle modifications than those with T1DM (78.4% vs. 21.6%, p=0.001). However, compared to people with T2DM, people with T1DM were more likely to adhere to a diabetes mellitus management plan that included insulin therapy and lifestyle modifications (73.7% vs. 26.3%, p=0.001). In addition, individuals with T2DM reported yearly eye exams at a significantly higher rate (57.1% vs. 42.9%, p=0.027) than those with T1DM. These findings are aligned with those of Keenum et al.'s findings in their study, which indicated that factors associated with higher adherence to follow-up care are being diagnosed with severe diabetic retinopathy and having a higher level of knowledge about the disease [16].

Nevertheless, the study revealed that following eye examination guidelines was associated with better diabetic retinopathy outcomes. Participants who reported being fully aware of the importance of eye examinations were more likely to have better control of glucose readings within the recommended range (56.8% vs 36.2%, p=0.017). They were also more likely to have had an eye examination specifically related to their diabetes within the previous year (62.8% vs 40.0% for those who were not aware, p=0.001). Participants who reported being fully aware of the importance of eye examinations were more likely to have heard about it from healthcare professionals (56.7% vs 17.6% for those who were somewhat or not at all aware, p=0.020). Individuals with T2DM who had an eye examination every year were more likely to understand the importance of eye examinations. This was demonstrated by 61.0% vs 25.0% for those who were unsure p=0.001) and were more likely to have had an eye examination within the previous year (58.3% vs 20.0% for those who were unsure, p=0.001). Reasons for not having an eye examination included a lack of awareness about the importance of eye exams (37.8%) and hospitals being too far away from their home (13.3%). To raise awareness and promote better retinopathy outcomes, healthcare professionals and public health campaigns must emphasize the importance of regular eye examinations in diabetics. These findings concur with Lee et al.'s findings in their study, which indicated that geographic location of the participants played a role in adherence to screening guidelines [17].

Some of the limitations encountered through the study was reliance on individuals' self-reported information, such the medical history, which can be influenced by memory lapses, failure to understand the question. However, the involvement of medical practitioners in data collection aided in mitigating these potential issues. Furthermore, the study only included people with diabetes who were attending a diabetes clinic. This may introduce selection bias and limit the findings' generalizability to the larger diabetic population. Finally, as the study being cross-sectional, it was difficult to establish a causal relationship between adherence to eye examination guidelines and glycemic control or retinopathy outcomes.

## Conclusions

In conclusion, this study discovered that a significant proportion of diabetics did not follow eye examination guidelines. The most common reason for not getting an eye examination was a lack of awareness about the importance of regular eye exams. Those who had had diabetes for more than 10 years and were fully aware of the serious eye complications and diabetic retinopathy were more likely to follow eye examination guidelines. Individuals with T2DM were also more likely to follow a management plan that included anti-diabetic medications and lifestyle changes, whereas those with T1DM were more likely to follow a management plan that included insulin therapy and lifestyle changes. Adherence to eye examination guidelines was associated with better glucose control and more frequent eye examinations for diabetes. These findings highlight the importance of integrating routine eye checkups into diabetes management plans during clinical visits.
